# Chronic insomnia disorder as risk factor for stroke: a systematic review

**DOI:** 10.1055/s-0042-1755227

**Published:** 2022-12-28

**Authors:** Luiz Augusto Soares Silva, Mateus Molin do Amaral, Vanise Grassi, André Luiz Rodrigues Palmeira

**Affiliations:** 1 Universidade do Vale do Taquari, Centro de Ciências Médicas, Lajeado, Brazil.

**Keywords:** Sleep Initiation and Maintenance Disorders, Risk Factors, Stroke, Sleep-Wake Disorders, Distúrbios do Início e da Manutenção do Sono, Fatores de Risco, Acidente Vascular Cerebral, Transtornos do Sono-Vigília

## Abstract

**Background** Stroke is one of the main causes of mortality worldwide. Nonetheless, there are still risk factors that have not been fully elucidated, such as chronic insomnia disorder.

**Objective** To evaluate the association between chronic insomnia disorder and the risk of stroke in adults, through a systematic review.

**Methods** Multiple studies available in the Embase, Lilacs, and Medline platforms were evaluated in English, Spanish, French, and Portuguese. The selection of papers was restricted to those that had investigated the association between chronic insomnia disorder and stroke in adults, regardless of gender or nationality, without a previous history of stroke. The data was extracted with the Cochrane Effective Practice and Organization of Care (EPOC) form. The risk of bias was evaluated by the EPOC Risk of bias tool.

**Results** A total of 138 articles were identified. After a detailed evaluation with the eligibility criteria, four articles were included in the present systematic review. Three of them recognized the association between chronic insomnia disorder and stroke. The comparative analysis was limited, since the studies used distinct insomnia classifications. Regarding the risk of bias, the analysis displayed an important risk in the selection and allocation of participants, besides the use of own insomnia diagnosis criteria, disrespecting chronology and factors indicated by already established classifications.

**Conclusions** There is not enough data to determine that chronic insomnia disorder is a risk factor for stroke. The present study points out the existence of a possible relationship between insomnia disorder and stroke, suggesting that further studies adopt standardized criteria and instruments.

## RESUMO

**Antecedentes** O acidente vascular cerebral (AVC) é uma das maiores causas de mortalidade do mundo. Ainda assim, existem fatores de risco não identificados, como o transtorno de insônia crônica.

**Objetivo** Avaliar a associação entre distúrbio crônico de insônia e o risco de AVC em adultos, por meio de uma revisão sistemática.

**Métodos** Foram avaliados estudos publicados em inglês, espanhol, francês e português, disponíveis nas plataformas Embase, Lilacs e Medline. Os estudos incluídos foram aqueles que investigaram a associação entre transtorno de insônia crônica e AVC em adultos, sem restrição de gênero ou de nacionalidade, sem história prévia de AVC. Os dados foram extraídos baseados no formulário da Cochrane Effective Practice and Organisation of Care (EPOC, na sigla em inglês). O risco de viés foi avaliado por meio da ferramenta EPOC Risk of bias tool.

**Resultados** Foram identificados 138 artigos, entre os quais, após avaliação detalhada para os critérios de elegibilidade, 4 foram incluídos na revisão sistemática. Destes, três encontraram associação entre insônia e AVC. Contudo, a análise comparativa apresenta limitações, pois nenhum dos estudos utilizou uma classificação comum de insônia. Quanto à análise do risco de viés, os estudos demonstraram um importante risco de viés na seleção e na alocação de participantes, além de utilizarem critérios diagnósticos de insônia próprios, sem respeitar cronologia e fatores indicados por classificações já estabelecidas.

**Conclusões** Não existem dados suficientes para determinar que o transtorno crônico de insônia se configura como fator de risco para AVC. A análise aponta para a existência de uma possível relação, sugerindo-se para estudos posteriores o uso de critérios e instrumentos padronizados.

## INTRODUCTION

Stroke constitutes one of the main causes of mortality worldwide, accounting for 6 million victims in 2016, 1 and it is estimated that 1 in 4 adults will have a stroke throughout their lifetime. 2 Epidemiologic studies that investigate the risk factors for cerebrovascular diseases point out a relation between sociodemographic features, family medical history, specific pathologies, and life habits with increased risk of stroke. Aging, male, black ethnicity, family history of cerebrovascular diseases, systemic arterial hypertension, diabetes mellitus, dyslipidemia, heart diseases, smoking, and alcoholism are some of the known risk factors. 3

The relationships between some possible risk factors with stroke were less investigated, among which stand out the sleep disorders (SDs). Previous studies suggest that chronic insomnia disorder may lead to metabolic abnormalities 4 and to an elevation of inflammatory cytokines, 5 among other aspects not fully elucidated, motivating an increasing number of research. Chronic insomnia is the most prevalent SD, present in ∼ 10% of the global population, representing a serious public health problem, impacting on quality of life and triggering or exacerbating other comorbidities.

Chronic insomnia disorder is characterized by difficulties in initiating or maintaining sleep and presence of nonrestorative sleep, with daytime dysfunction. 6 It has several consequences, such as fatigue, depressed mood, irritability, malaise, and cognitive impairment. 7 Based on established clinical criteria, these symptoms must occur at least 3 times in a week and for at least 3 months. 8 Since the diagnosis of chronic insomnia disorder is predominantly clinical, the impact of this SD must be investigated. Aiming to complement this evaluation, standardized instruments such as the Pittsburgh Sleep Quality Index (PSQI) and the Epworth Sleepiness Scale (ESS) may be used. 9 The PSQI evaluates the sleep quality in the past month, seeking to evaluate dysfunctional patterns and the severity of the problem in the present, so that a score ≥ 5 is an indicative that the sleep quality of the individual is getting worse. 10 The ESS graduates the probability of the patient falling asleep performing daily activities; to be considered as suffering from excessive daytime sleepiness, the patient must have a global score ≥ 10. 11

Considering the importance of controlling modifiable risk factors to prevent cerebrovascular events 12 and that chronic insomnia disorder might be included among these, the investigation of the relationship between insomnia and stroke is an important field of scientific research. The aim of the present study is to evaluate the association between chronic insomnia disorder and stroke.

## METHODS

The present study is a systematic literature review based on a qualitative analysis of publications and its synthesis in a table with the main findings.

### Database and research

The Embase, Lilacs, and Medline electronic databases were searched. The following Emtree terms were used in the Embase research: *insomnia* and *cerebrovascular disorders* . In the Medline research, the following Medical Subjective Headings (MeSH) terms were used: *cerebrovascular disorders* and *sleep initiation and maintenance disorders.* In the Lilacs research, the search terms were *AVC* and *insônia* . The research included all relevant studies published before 10 March 2020, limited to English, French, Spanish, and Portuguese.

### Study selection

The considered studies were observational, both cross-sectional and longitudinal. The included studies were those that investigated the association between chronic insomnia disorder and stroke. Furthermore, they needed to meet the following criteria: (1) population consisting of adults (≥ 18 years old); (2) both genders; (3) no nationality restriction; (4) without previous diagnosis of stroke. As exposure, we considered patients with insomnia symptoms, such as: (1) difficulties in initiating or maintaining sleep; (2) presence of nonrestorative sleep, with daytime dysfunction; (3) early morning awakening; (4) the symptoms must happen at least 3 times in a week, for at least 3 months. For the control group, patients without the previously related conditions were considered (Pittsburgh scale < 5 or Epworth scale < 10).

The outcomes considered were those that presented total incidence of total stroke, the incidence and mortality of ischemic and/or hemorrhagic stroke. Also, stroke severity (as evidenced by the National Institutes of Health Stroke Scale [NIHSS] 13 and the Modified Rankin Scale [MRS]) 14 was evaluated.

Two reviewers (Silva L. A. S. and Amaral M. M.) independently screened the studies based on their titles and abstracts, evaluating their eligibility. After this first step, the remaining studies were fully analyzed for eligibility, according to the established criteria. In cases of disagreement regarding the eligibility of the study in any step, other two reviewers (Palmeira A. L. R. and Grassi V.) were consulted.

### Data extraction

Two reviewers (Silva L. A. S. and Amaral M. M.) extracted the data independently and in duplicate, using the standardized form from the Cochrane Effective Practice and Organisation of Care (EPOC). 15 The reviewers were previously trained to perform the data extraction – some modifications were made to the form aiming to facilitate the review process.

Relevant information was collected regarding the study design, participants (number and epidemiologic/demographic characteristics), and a full description of the intervention performed. Details regarding the outcomes were also extracted (including a description of how and when the outcomes were measured).

### Risk of bias

The EPOC Risk of bias tool 15 was used to evaluate the risk of bias in all studies. This tool includes five domains of bias: selection bias, performance bias, detections bias, attrition bias, and reporting bias. Other potential threats to the study validity that were not included in the previous domains were included in the domain “others.” Two reviewers (Silva L. A. S. and Amaral M. M.) evaluated the risk of bias in duplicate and independently; in case of disagreement, a third reviewer (Grassi V.) was consulted to resolve the issue.

## RESULTS

### Database research

**Figure 1.**
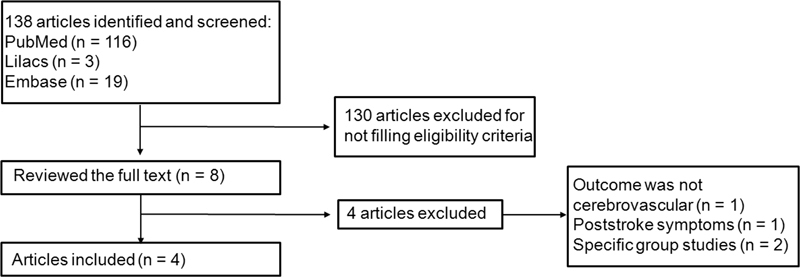
Flow diagram of the study selection process.

In the studies considered eligible to the analyses, none of the preconized tools for the evaluation of insomnia were used. In a post-hoc analysis performed on March 10, 2020, in which the articles were read in full to evaluate the eligibility criteria, 138 articles were identified and 8 16 17 18 19 20 21 22 23 articles were selected for a detailed evaluation. After this analysis, four 16 17 18 19 articles were included in the systematic review. A flow diagram of the selection process of the present study is shown in Figure 1 .

### Studies and population characteristics

Among the four articles selected for the present review, one was performed with Chinese 16 participants, one was performed with German 17 participants, and two 18 19 were performed with Taiwanese participants. The analyzed studies were based on population databases, which contain information on the sleep of the participants, whose insomnia symptoms were obtained from forms applied by health professionals. The median follow-up duration was 9,8 years, ranging from 4 to 14 years. The median age of the participants was 50,7 years old.

The studies evaluated adults (> 18 years old) from both genders without a previous diagnosis of stroke, 16 17 18 19 coronary heart diseases, 16 18 cancer, 16 or sleep apnea ( Table 1 ). 18 19

**Table 1:** Synthesis of the qualitative analysis

Author (year)	Sample ( *n* )	Study population	Definition of insomnia	Outcomes	Results
Zheng (2019) 16	487,200	Adults (30–79 years old), without a previous diagnosis of stroke, coronary heart disease, or cancer.	Difficulties in initiating or maintaining sleep, early morning awakening, and daytime dysfunction; for at least 3 days/week in the past month.	Risk of total stroke, hemorrhagic stroke, and ischemic stroke. Associations of any insomnia symptoms and number of insomnia symptoms with risk of stroke.	All three symptoms (DIMS, EMA, and DDF) were associated with slightly increased risks of total stroke incidence (HR 1.05, 1.05, and 1.08; *p* < 0.05) and ischemic stroke incidence (HR 1.06, 1.07, and 1.09; *p* < 0.05). No associations were observed between the 3 symptoms and hemorrhagic stroke incidence. Compared with those with no insomnia symptoms, 1, 2, or 3 symptoms increases the risk of stroke by 7, 10, and 18%, respectively.
Helbig (2015) 17	15,746	Adults (25–74 years old) without a previous diagnosis of stroke.	Difficulties in initiating or maintaining sleep, considering a frequency chosen as “often”.	Incidence of total stroke, fatal stroke, and nonfatal stroke.	Symptoms of insomnia were not significantly predictive of the incidence of total strokes, nonfatal strokes, and fatal strokes in either gender.
Hsu (2015) 18	44,080	Adults (≥ 20 years old), without a previous diagnosis of stroke, coronary heart disease, or sleep apnea.	Difficulties in initiating or maintaining sleep, according to the ICD-9-CM.	Incidence of total stroke and risk of developing stroke.	Individuals with insomnia had a higher incidence of stroke (8.01 versus 3.69 per 1,000 individuals/year; *p* < 0.001). Insomnia was independently associated with an increased risk of developing stroke (HR 1.85; *p* < 0.001).
Wu (2014) 19	85.752	Adults (≥18 years old), without a previous diagnosis of stroke or sleep apnea.	Difficulties in initiating or maintaining sleep, according to the ICD-9-CM.	Incidence of total stroke, hemorrhagic stroke, and ischemic stroke. Comparison of assigned risk of stroke between patients with and without insomnia.	The incidence rate of stroke was significantly higher (IRR 1.85) in insomniacs than in noninsomniacs. The distributions of stroke subtypes were: ischemic stroke (IRR 1.79) and hemorrhagic stroke (IRR 1.32). Those with insomnia were at increased risk of stroke by a magnitude of 54% (HR 1.54).

Abbreviations: DDF, daytime dysfunction; DIMS, difficulties in initiating or maintaining sleep; EMA, early morning awakening; HR, hazard ratio; ICD-9-CM, International Classification of Diseases, Ninth Revision, Clinical Modification; IRR, incidence rate ratio.

### Chronic insomnia disorder definition

The insomnia definition was different for all analyzed studies. For Zheng et al., 16 the participants were considered as having chronic insomnia disorder if they presented difficulties in initiating or maintaining sleep, early morning awakening, daytime dysfunction, and the symptoms must occur for at least 3 days in a week for the last month. Helbig et al. 17 considered that participants with chronic insomnia disorder were those who chose “often” as the answer to the question about in which frequency they had difficulties in initiating or maintaining sleep.

Regarding the studies by Hsu et al. 18 and Wu et al., 19 the definition of chronic insomnia disorder for both studies was based on the International Classification of Diseases, Ninth Revision, Clinical Modification (ICD-9-CM). Patients codified with 780.52 (insomnia, unspecified), 307.41 (transient disorder of initiating or maintaining sleep), or 307.42 (persistent disorder of initiating or maintaining sleep) were considered the group of patients with insomnia.

### Outcomes

The association between insomnia symptoms and the incidence/risk of stroke was the common outcome among the studies ( Table 1 ). The study by Zheng et al. 16 reported the risk of total stroke and its subtypes and performed a comparison of any insomnia symptom and of the number of insomnia symptoms with the risk of stroke. The three symptoms were: difficulties in initiating or/and maintaining sleep, early morning awakening, and daytime dysfunction. These symptoms were associated with slightly increased of risks of total stroke, hazard ratio (HR), confidence interval 95% (IC 95%), 1.05 (1.02–1.08), 1.05 (1.02–1.08), and 1.08 (1.02–1.14), respectively, *p* < 0.05. There was an increase in the risk of ischemic stroke HR 1.06 (1.03–1.10), 1.07 (1.04–1.10) and 1.09 (1.02–1.16), *p* < 0.05. There was no association with hemorrhagic stroke. According to the study, when comparing patients without any symptom of insomnia with those who presented symptoms, there was an increase in the risk of stroke of at least 7% for 1 symptom, and at most 18% for 3 symptoms.

The study by Helbig et al. 17 evaluated the association between chronic insomnia disorder and total stroke incidence, as well as the incidence of fatal and nonfatal stroke, but no significant associations were found.

Meanwhile, the study by Hsu et al. (2015) 18 evaluated the incidence of total stroke and the risk of developing future stroke. The incidence of total stroke in the group with participants with chronic insomnia disorder was 8.01 per 1,000 individuals/year, approximately double of the incidence in the group without insomnia (3.69 per 1,000 individuals/year; *p* < 0.001). Furthermore, the study reports that patients with chronic insomnia disorder presented an increased risk of developing a future stroke (HR: 1.85, 95%CI: 1.62–2.12; *p* < 0.001).

The study by Wu et al. 19 evaluated outcomes involving the total incidence of stroke in patients with chronic insomnia disorder, the incidence of ischemic and hemorrhagic stroke, and a comparison of increased risk of stroke between patients with and without chronic insomnia disorder. The total incidence of stroke in patients with chronic insomnia disorder were significantly higher (incidence rate ratio [IRR]: 1.85; 95%CI: 1.67–2.05). According to the subtypes, the following data were found ischemic stroke (IRR: 1.79; 95%CI: 1.56–2.06) and hemorrhagic stroke (IRR: 1.32; 95%CI: 1.02–1.68). Those with chronic insomnia disorder were at increased risk of stroke by a magnitude of 54% (HR: 1.54).

### Risk of bias analyses

**Figure 2.**
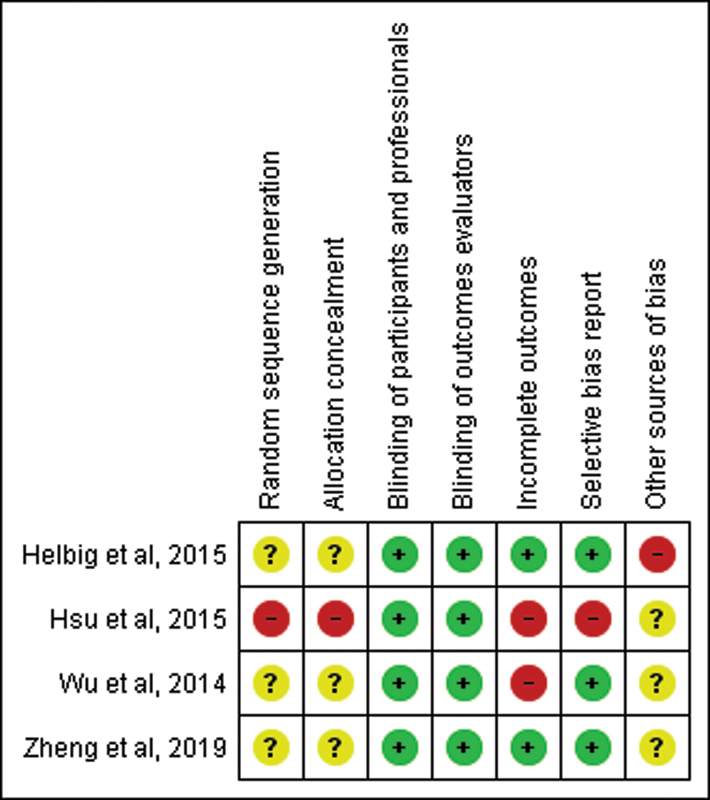
Risk of bias summary. Review the judgement of the authors of each risk of bias item for each included study. These items were classified as 'adequate' (low risk of bias) with +, 'inadequate' (high risk of bias) with -, or 'unclear' with ?.

The studies presented an important risk of selection and allocation bias. The other risk of bias identified were the criteria for the diagnosis of chronic insomnia disorder. The assessment of the risk of bias is presented in Figure 2 .

## DISCUSSION

In the present systematic review, among the 4 included studies, 3 indicated an association between chronic insomnia disorder and stroke. The definition and evaluation of chronic insomnia disorder in each study were different, mainly regarding the frequency of the symptoms, a situation found in previous studies. 24 Thus, the importance of the universalization and unification of the definition of chronic insomnia disorder arises, mainly in the execution of observational studies, so that it is possible to equate and quantify the results found among studies.

The International Classification of Sleep Disorders – Third Edition (ICSD-3), 8 published by American Academy of Sleep Medicine in 2014, defines chronic insomnia disorder as dissatisfaction with the sleep quality or quantity associated with nocturnal symptoms (sleep initiation and maintenance disturbance, early morning awakening), a situation that causes daytime dysfunction, which includes fatigue, sleepiness, decreased mood, cognitive impairment, and even social impairment. To meet these classification criteria, these symptoms must be present for at least, 3 months and occur at least 3 times per week. In this case, it becomes evident that none of the reviewed studies followed a common classification, neither respected the chronology supported by these classifications.

Another well-known classification of chronic insomnia disorder is the one presented by the Diagnostic and Statistical Manual of Mental Disorders - Fifth Edition (DSM-V), 25 which defines chronic insomnia disorder in a quite similar way as the ICSD-3. Although the classifications are highly similar, the one established by the DSM-V considers important exclusion criteria, such as the effect of substances and other mental or physical conditions. On the other hand, the ICSD-3 8 defines chronic insomnia disorder including the criteria of resistance to going to bed and the difficulty in sleeping without a parent. Nevertheless, the found differences between the classification are minimal, so both classifications can be used to diagnose chronic insomnia disorder. Therefore, their use is indicated to facilitate the comparison of future research results.

None of the analyzed studies used the Pittsburgh 10 and Epworth 11 scales as evaluation tools. Through the years, these scales proved to be effective methods to evaluate SDs, 26 27 28 29 mainly insomnia, since they allow to quantify and qualify the sleep evaluation. Thus, even though they are not widely used in clinical practice, they have a large potential to, in the future, establish thresholds to relate quantitatively SDs with other pathologies, such as stroke. We emphasize the importance of using these tools in clinical practice and in scientific research, so the diagnosis and classification of SDs can be unified and universalized, with the idea of being more understood in all its dimensions. In the studies mentioned in the present paper, no other insomnia scales were described.

In addition to the cited tools, the Athens Insomnia Scale 30 (AIS) and the Insomnia Severity Index (ISI) could be used; 31 both tools are not yet validated in Brazil. The first one evaluates difficulties referring to the induction and maintenance of sleep and early morning awakening, besides having questions about sleep quality and the presence of daily sleepiness, being able to measure the impact of SDs by considering the symptoms that must occur at least three times in a week in the last month. 30 The second one analyses the severity of the presented symptoms in chronic insomnia disorder, including the perception of sleep changes through the days, the preoccupation of the patient about the symptoms, and the satisfaction and the impact of chronic insomnia disorder in the dairy activities, considering the symptoms that occurred in the last month. 31

The concomitant prevalence of other comorbidities, such as diabetes mellitus, arterial hypertension, dyslipidemia, well-known and consolidated risk factors for stroke, 32 was significant. Also, it is important to mention that chronic insomnia disorders increase the likelihood of developing these comorbidities. In this context, the isolated analyses of chronic insomnia disorder as a risk factor for stroke is a challenge, for none of the studies found in the searched databases considered the existence of other risk factors, such as smoking, alcoholism, and sedentary lifestyle. Another raised hypothesis is that patients present symptoms of chronic insomnia disorder and other SDs before other cerebrovascular risk factors are identified. Thus, the early tracking of chronic insomnia disorder might represent a tool of primary prevention.

The present study has some important limitations that must be noted. In the chronic insomnia disorder diagnosis, heterogeneity in the inclusion criteria was noted, based in the patient report, without application of specific criteria. Regarding insomnia severity, none of the preconized scales (PSQI and ESS) was used in the studies, making it difficult to estimate the impact of insomnia in the incidence of stroke.

Two previous meta-analyses are pivotal in this discussion regarding the relationship between chronic insomnia disorder and stroke. Ge et al. 33 studied the relationship between sleep duration and stroke incidence, finding that both short (< 4 to 6 hours) and long (> 8 to 10 hours) sleep durations increase the risk of stroke (odds ratio [OR]: 1.71 and 2.12, respectively). Nevertheless, chronic insomnia disorder symptoms were not assessed in this analysis. He et al. 24 investigated the relationship between insomnia symptoms and cardiocerebrovascular events and included 15 publications (23 cohorts) in this analysis, demonstrating an increased risk about initiating (relative risk [RR] ± 1.27) and maintening (RR ± 1.11) sleep disorder, as well as with nonrestorative sleep (RR = 1.18). It is noteworthy, however, that only two studies, one included in our analysis, 17 used stroke as an outcome in its meta-analysis.

An European Academy of Neurology (EAN), European Respiratory Society (ERS), European Sleep Research Society (ESRS), and European Stroke Organization (ESO) statement 34 points out that the risk of stroke associated with chronic insomnia disorder is still uncertain and emphasizes the variability between the used diagnostic criteria, mainly based on subjective criteria. Adoption of standardized, validated and more objective evaluation scales are necessary in future studies, as well as better assessment of comorbidities. Also, repeated measures are necessary, since insomnia symptoms may change over time.

Despite the aforementioned challenges in executing the present systematic review, we were able to contemplate chronic insomnia disorder in an attempt to relate it to an increased risk of stroke. At this moment, there is not enough data to determine that chronic insomnia disorder is a risk factor for stroke. The analysis points out the existence of a possible relationship that must be studied with better criteria. We suggest that the screening of chronic insomnia disorder be included in the research of comorbidities precociously, through uniformized diagnosis criteria. We also indicate the utilization of the PSQI and ESS to objectively evaluate and quantify the symptoms of insomnia and their repercussions in the quality of life of patients. To solve the loss of the comparative capacity of the studies, generated by multiples and different definitions, we suggest the application of one of the cited classifications (ICSD-3 and DSM-V).
